# Performance analysis of an orbital angular momentum multiplexed amplify-and-forward radio relay chain with inter-modal crosstalk

**DOI:** 10.1098/rsos.181063

**Published:** 2019-01-23

**Authors:** B. Allen, D. Simmons, T. D. Drysdale, J. Coon

**Affiliations:** 1 Network Rail, The Quadrant: MK, Elder Gate, Milton Keynes MK9 1ER, UK; 2 Department of Engineering Science, University of Oxford, Parks Road, Oxford OX1 3PJ, UK; 3 School of Engineering, University of Edinburgh, Edinburgh EH9 3FG, UK

**Keywords:** wireless communications, relay chain, orbital angular momentum multiplexing, spectral efficiency

## Abstract

The end-to-end spectral efficiency and bit error rate (BER) of an amplify-and-forward (AF) radio relay chain employing orbital angular momentum (OAM) multiplexing is presented. The inherent divergence of a beam carrying OAM is overcome by means of a lens. Modelled and measured inter-modal crosstalk levels are incorporated into the analysis. The results show that an end-to-end spectral efficiency of up to 8 bits s^−1^ Hz^−1^ is achievable using four OAM modes to multiplex four parallel data streams over 20 hops, provided that the detrimental effects of inter-modal crosstalk are mitigated. The spectral efficiency is expected to scale further by using more OAM modes. The BER profile along the relay chain is analysed for each of the four OAM modes.

## 1. Introduction

Relay chains are well known as a means of improving the reach of wireless networks [1,2], for example, when serving as a data backbone along transportation corridors. With the emergence of intelligent transport and the enduring demand for broadband connectivity while on the move, conventional mobile networks are struggling to provide the required coverage and capacity. The emergence of 5G has stimulated the development of millimetre-wave wireless devices for carrying high-capacity data services over short ranges such as between ground and vehicle. Ground nodes will need connecting by means of a suitable data backbone. In this context, a relay chain of millimetre-wave links is one means of improving the reach of such networks. However, conventional millimetre links are unlikely to be able to provide the required capacity for supporting the aggregate data-rate along core data backbones, where it is thought that data rates of at least 40 Gb s^−1^ will need to be accommodated [3].

**Figure 1.**
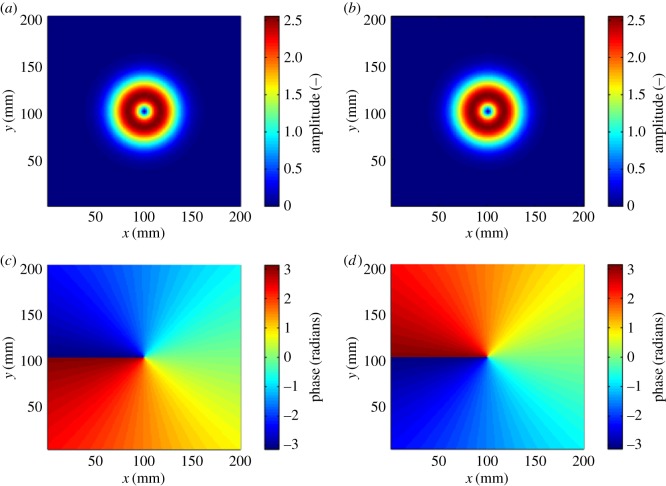
Amplitude (*a,b*) and phase (*c,d*) profiles of LG modes l = +1 (*a,c*) and l = −1 (*b,d*).

**Figure 2.**
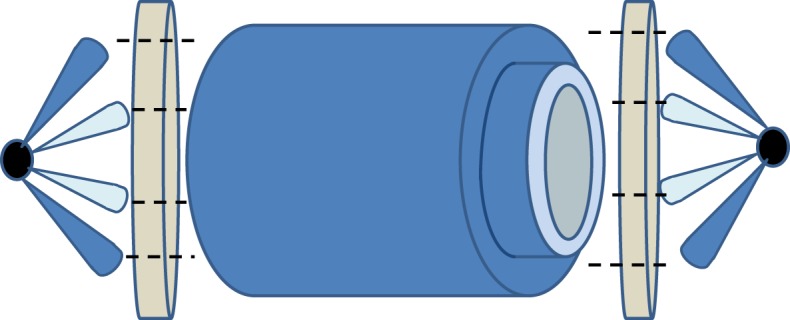
Illustration of the effect of collimating lens on two divergent OAM beams.

Currently, orbital angular momentum (OAM) radio is attracting attention as a means of significantly increasing the capacity of line-of-sight radio links [4–7]. OAM radio can be considered as a form of multiple input, multiple output (MIMO) communications that relies on spatial diversity for attaining a performance gain [8], but in contrast with conventional MIMO implementations, it is suited to line-of-sight links. As is the case for MIMO, it has the potential to support several parallel data streams by sending each stream on a separate ‘mode’. In this case, a mode relates to the radiated spatial amplitude and phase patterns as defined by the family of Laguerre–Gaussian (LG) beams [9], where the phase signatures are mutually orthogonal [10]. Owing to the phase trajectory of each mode having a ‘twist’, they are shown to have a topological charge and hence referred to as OAM modes [11]. An example ideal amplitude and phase patterns of modes ±1 are shown in figure 1. The amplitude profiles are identical and exhibit a ‘vortex’ in the centre. The effect of the vortex is that the beam is heavily divergent, as rigorously described in [12]. This may be mitigated by means of placing a collimating lens such as the ones described in [13,14] in the path of the beam. An ideal collimating lens would transpose the divergent beams into concentric rings with radiuses increasing with topological charge. This is illustrated in figure 2. The phase profiles in figure 1 show a linear increment of phase around the central discontinuity in clockwise and counter-clockwise directions, respectively. OAM modes differ from polarization in that polarization may be likened to spin angular momentum (SAM), which is an independent effect to OAM [15]. Polarization multiplexing is a well-known phenomenon [16] which may be trivially added to further enhance the spectral efficiency of an OAM radio system. The focus of this paper is the exploitation of OAM modes as a means of providing an additional degree of freedom that may be exploited independently. There are two benefits to adopting additional degrees of freedom in this way: first, increased total transmission power without the inefficiency of combining the power of multiple millimetre-wave sources in a single-mode link and, second, increased spectral efficiency for the same total transmission power compared to a single-mode link. Ideally, each OAM mode is independent of the others, but in practice a level of inter-modal crosstalk occurs between each mode is present because of coupling of RF signal paths, generator and detector imperfections, multipath caused by poor link positioning or close-by objects causing field perturbations. This will impact the achievable channel capacity available along the relay chain. Note that OAM radio link analysis may be expressed in matrix form with the channel matrix comprising eigenmodes, in the same way as MIMO links can be analysed [8].

This paper presents, to the best of our knowledge, the first theoretical analysis of an OAM radio relay chain, including the effect of inter-modal crosstalk. Crosstalk is incorporated from measured data and by means of a simple model which is proposed. A linear chain of amplify-and-forward (AF) relays is considered, with OAM modes used as a ‘bearer’ for multiplexing independent data streams along the chain. The performance is considered in terms of spectral efficiency along the chain (measured in bits s^−1^ Hz^−1^) and bit error rate (BER).

The following section presents the analytical model used to determine end-to-end spectral efficiency of such an OAM radio relay network. Spectral efficiency and BER results for a chain with four OAM modes are then determined for the cases of: zero crosstalk, crosstalk modelled using a power law profile, and measured crosstalk profiles, as well as for a relay chain without OAM multiplexing. The results are discussed and then conclusions drawn.

## 2. System model

**Figure 3.**
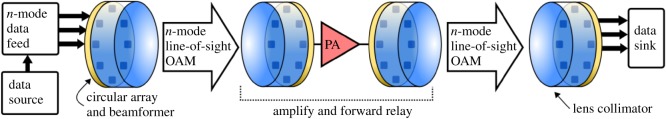
Block diagram of an OAM radio AF relay chain with a data source, relay, antenna array, beamformer, collimating lens and data sink.

We consider an *n*-hop AF OAM-based radio relay chain, as depicted in figure 3. A data source generates data to be distributed equally between the OAM modes for transmission by means of a multiplexer, which may be implemented as a fixed beamforming network for each OAM mode. We assume here that the beamformers are configured to produce OAM modes of ±1 and ±2. The four data streams are fed to a circular antenna array, such as the one described in [17], which is configured to generate the four OAM modes. The array transmits and receives through a lens such as that described in [13]. As shown in [13], the lens is necessary for collimating the OAM radio beam to mitigate the inherent beam divergence evident in the array radiation characteristics when observed without a collimating lens. This enables practical link distances to be achieved, which are otherwise not possible due to beam divergence.

Each relay node consists of a receive lens/antenna array combination, power amplifier (PA) and transmit antenna array/lens. For this paper, the PA is assumed to be operating in its linear region, of fixed gain and a source of Gaussian noise. A line-of-sight link with no multipath is also assumed and is considered representative due to the use of highly directional antennas, as is common practice in millimetre-wave links.

We consider an *i*-mode OAM scheme across each hop. This is with equal power allocated to each mode, i.e. water-filling is not considered. The analysis considers a normalized system, in that the actual separation distance between relay nodes is unity. The analysis may, however, be readily extended to evaluate the impact of relay separation, path-loss and other link budget effects. It is known that the signalling model for an OAM system is a special case of MIMO signalling [8]. Consequently, we can recursively model the transmitted signal, *X*, at the *n*th relay as

2.1 $$X_{n}^{\left(\alpha \right)}=\alpha_{n}(\xi X_{n-1}^{\left(\alpha \right)}+V_{n}),$$

where *ξ* describes the 4 × 4 channel matrix for each of the modes, *α_n_* describes the amplification factor applied at the *n*th relay and ***V****_n_* describes the noise vector associated with the reception/transmission of the signal from the *n*th relay. We can now model the spectral efficiency of the *i*th mode at the *n*th relay of the OAM relay chain [1] as

2.2 $$\eta_{n,i}=\frac{1}{2}\log\left(1+E_{i}(R_{I,n}^{\left(\alpha \right)}{\left(R_{N,n}^{\left(\alpha \right)}\right)}^{-1})\right),$$

where

$$\begin{array}{rl} R_{I,n}^{\left(\alpha \right)} & =\left(\;p_{0}\overset{n}{\underset{i=1}{\prod }}\alpha_{i}^{2}\right)\frac{\xi }{∥\xi {∥}_{F}}\cdots \frac{\xi }{∥\xi {∥}_{F}}\frac{\xi^{T}}{∥\xi {∥}_{F}}\cdots \frac{\xi^{T}}{∥\xi {∥}_{F}}\\ \mathrm{and}\;\;\;\;\;R_{N,n}^{\left(\alpha \right)} & =n_{0}\left(I_{4}+\overset{n}{\underset{l=2}{\sum }}\overset{n}{\underset{i=l}{\prod }}\alpha_{i}^{2}\frac{\xi }{∥\xi {∥}_{F}}\cdots \frac{\xi }{∥\xi {∥}_{F}}\frac{\xi^{T}}{∥\xi {∥}_{F}}\cdots \frac{\xi^{T}}{∥\xi {∥}_{F}}\right) \end{array}$$

are the autocorrelation matrices of the received information and noise vectors at the *n*th node, *I*_4_ represents the 4 × 4 identity matrix, *ξ_i_* (.) is the *i*th eigenvalue of the matrix denoted in the brackets, *n*_o_ is the noise variance at node zero, and *p*_o_ is the transmit power at node zero. The normalization factor *‖ξ‖_F_* represents the Frobenius norm of *ξ* and is included to ensure a fair comparison between each of the crosstalk scenarios considered below. For zero crosstalk, *ξ* is an identity matrix. In practice, crosstalk is introduced because of imperfections in alignment, nearby objects and coupling between antennas, cables and devices. The consequence is that the power in one OAM mode is partially spread into other modes, hence causing interference to data transmitted on the other modes. We assume *ξ* to be one of the following matrices and correspond to a four-mode OAM scheme employing modes ±1 and ±2.

2.3 $$\xi_{\mathrm{Id}}=\left[\begin{array}{cc} \begin{array}{cc} 1 & 0\\ 0 & 1 \end{array} & \begin{array}{cc} 0 & 0\\ 0 & 0 \end{array}\\ \begin{array}{cc} 0 & 0\\ 0 & 0 \end{array} & \begin{array}{cc} 1 & 0\\ 0 & 1 \end{array} \end{array}\right],$$

2.4 $$\xi_{\mathrm{Mo}}=\left[\begin{array}{cc} \begin{array}{cc} 0.986 & 0.608\\ 0.608 & 0.986 \end{array} & \begin{array}{cc} 0.375 & 0.231\\ 0.231 & 0.375 \end{array}\\ \begin{array}{cc} 0.375 & 0.231\\ 0.23 & 0.608 \end{array} & \begin{array}{cc} 0.986 & 0.608\\ 0.375 & 0.986 \end{array} \end{array}\right]$$

2.5 $$\mathrm{and}\;\;\;\;\;\;\;\;\xi_{\mathrm{Me}}=\left[\begin{array}{cc} \begin{array}{cc} 1.000 & 0.530\\ 0.410 & 1.000 \end{array} & \begin{array}{cc} 0.316 & 0.158\\ 0.141 & 0.721 \end{array}\\ \begin{array}{cc} 0.588 & 0.404\\ 0.241 & 0.737 \end{array} & \begin{array}{cc} 1.000 & 0.182\\ 0.333 & 1.000 \end{array} \end{array}\right].$$

Equation (2.3) corresponds to the scenario in which no crosstalk is present. In (2.4), the rows have been modelled using an exponential model such that it results in a matrix similar to the measured profile in (2.5). Note that using a model with more degrees of freedom would result in a closer match between the measured and modelled values. The exponential model has been adopted here for convenience, and a detailed study of crosstalk models is beyond the scope of this paper. The chosen model has the format *y = m.*e*^bx^*, where e is the exponential function; *b* is the exponent selected to be −0.484; *m* is a constant selected to be 1.6; *x* is the distance from the central diagonal; and *y* is the resulting value. *m* and *x* were selected through trial and error such that the modelled curve closely followed the measured values.

The measured values of crosstalk in (2.5) are those obtained from an experimental OAM radio system operating at 5.9 GHz while inside an anechoic chamber [3]. The transmit array was an eight-element circular array of diameter 180 mm with programmable digital phase shifters for configuring the OAM mode. The insertion losses of the eight transmit channels matched to within a range of 0.6 dB. The receiver was a four-element partial aperture receiver with programmable phase shifters to select the desired mode, as described in [3]. A partial aperture design was used to reduce the overall size of the receive antenna to an arc of 187° on a diameter of 1 m. The antenna array would be smaller if we had used lenses, but no lenses were available at the time of the experiment. At millimetre-wave frequencies, it is practical to produce lenses from dielectric materials, but such lenses would be prohibitively expensive to produce at the operating frequency of our present experiment [14]. Nonetheless, the results we obtain represent a useful, if slightly pessimistic, benchmark for the practical performance of a multi-mode OAM system. The receive antenna was located 2.37 m from the transmitter, placing it in the far-field (the far-field for this transmitter begins at 1.27 m). The received signal power for each of the 16 modal combinations was normalized and used to construct (2.5). Normalization was by dividing each column of the matrix of the received power matrix by the maximum value of that column.

## 3. Analysis and results

### 3.1. Spectral efficiency

**Figure 4.**
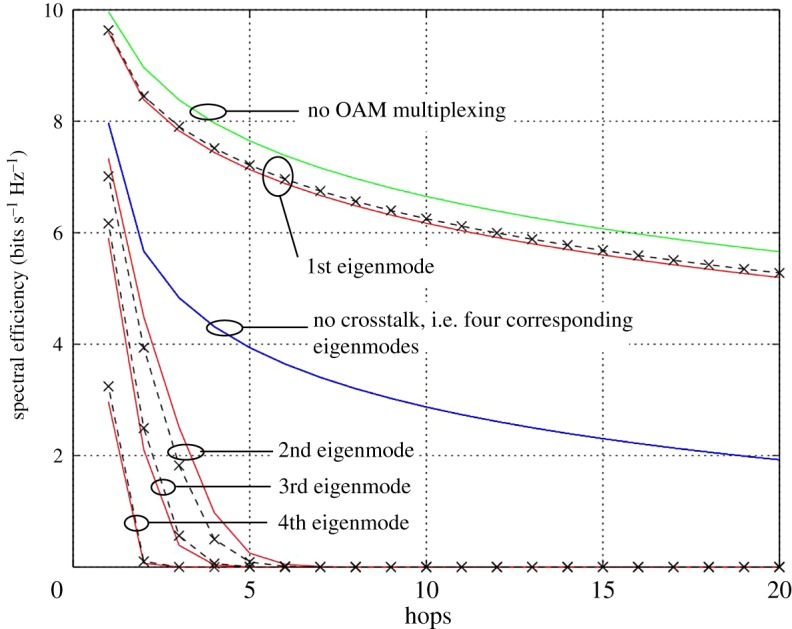
Spectral efficiency of an AF relay chain for each mode (i) without OAM multiplexing (green curve), (ii) with no crosstalk (blue curve), (iii) modelled crosstalk (black curve) and (iv) measured crosstalk (red curve).

**Figure 5.**
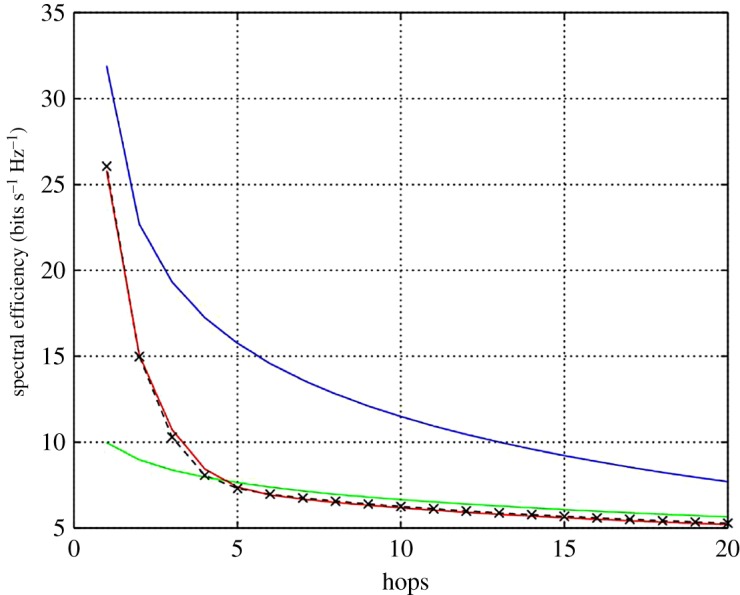
Total spectral efficiency of an AF relay chain (i) without OAM multiplexing (green curve), (ii) with no crosstalk (blue curve), (iii) modelled crosstalk (black curve) and (iv) measured crosstalk (red curve).

**Figure 6.**
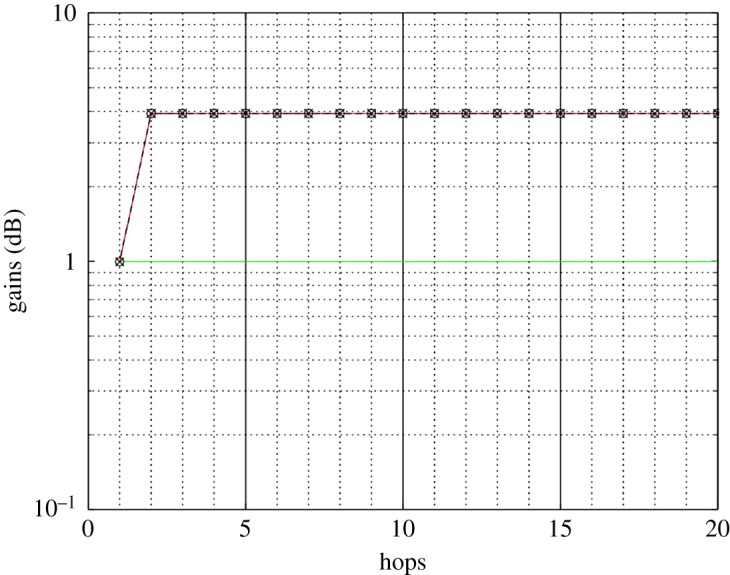
AF relay gain versus number of hops (i) without OAM multiplexing (line with circle symbol), (ii) with no crosstalk (green line), (iii) modelled crosstalk (line with crosses), and (iv) measured crosstalk (red line).

Equation (2.2) has been evaluated over 20 hops in order to show the spectral efficiency profile along the relay chain for an average first hop signal-to-noise ratio (SNR) of 30 dB for each OAM mode. The resulting spectral efficiency profiles are shown in figure 4 for the case of a conventional AF relay chain, i.e. a single antenna non-OAM relay chain, and the three crosstalk scenarios relating to (2.3)–(2.5) and the corresponding OAM scheme. Each curve in figure 4 corresponds to the spectral efficiency relating to each of the eigenmodes determined from the channel matrix. Figure 5 is similar to figure 4, except that it shows the total spectral efficiency by summing those of each corresponding mode. The quasi-analytical results have been normalized so that the noise has unit power. In this work, to ensure a fair comparison, we have selected the relay gains, *α_n_*, so that the normalized transmitted power for all relays are the same. The resulting gain profile that is required along the relay chain to attain the required transmit power is shown in figure 6 for each crosstalk scenario.

### 3.2. Bit error rate

The above analysis is extended here to determine the bit error rate (BER) for each mode along the relay chain. Binary phase shift keying (BPSK) is assumed, where the BER is given as a function of SNR and *E_b_*/*N_o_* using expression (3.1) [18], where *E_b_* is the energy per bit and *N_o_* is the spectral noise density and erfc is the complimentary error function. Analysis for other modulation schemes may be conducted by replacing (3.1) with the appropriate expression for the desired modulation scheme; see, for example, [18] for other such expressions. Note that using eight-frequency shift keying (8-FSK), for example, improves the BER performance due to having a lower SNR requirement compared to BPSK; however, 8-FSK requires a wider transmission bandwidth when compared with that of BPSK.

3.1 $$\mathrm{BE}R_{\mathrm{BPSK}}=\frac{\mathrm{erfc}\left(\sqrt{\mathrm{SNR}}\right)}{2}=\frac{\mathrm{erfc}\left(\sqrt{E_{b}/N_{o}}\right)}{2},$$

SNR is determined in the usual way and extracted from the evaluation of equation (2.2):

3.2 $$\mathrm{SNR}=\frac{\mathrm{Signal}\;\;\mathrm{power}}{\mathrm{Noise}\;\mathrm{power}}.$$

**Figure 7.**
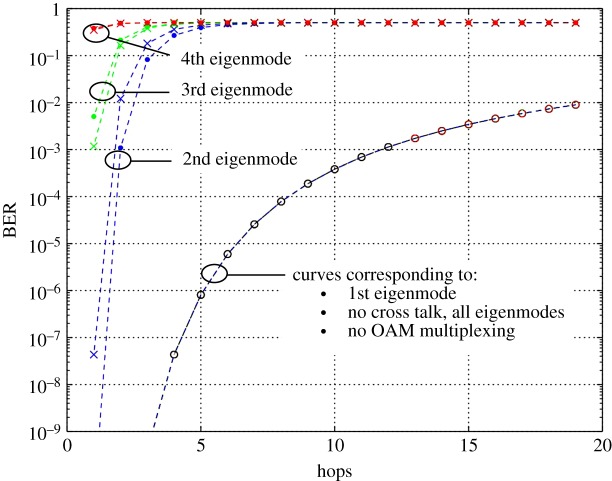
BER versus number of hops (i) without OAM multiplexing (continuous line), (ii) with no crosstalk (line with circle), (iii) modelled crosstalk (line with crosses) and (iv) measured crosstalk (line with filled circle).

Figure 7 shows the BER versus hops. Curves are plotted for the cases of when no OAM modes are used, four OAM modes with no crosstalk, four OAM modes with the measured crosstalk and four OAM modes with the modelled crosstalk.

## 4. Discussion

With reference to figures 4 and 5, the total spectral efficiency for each of the crosstalk scenarios and for a particular hop is the summation of the spectral efficiency of all corresponding individual eigenmodes. Figure 4 shows the spectral efficiency for the individual eigenmodes, whereas figure 5 shows the summation. For the case of no crosstalk, the four eigenmodes from the resulting channel matrix are identical and show a total of 32 bits s^−1^ Hz^−1^ is achievable at the start of the relay chain and reducing to 8 bits s^−1^ Hz^−1^ after 20 hops. For the case of a relay chain not employing OAM multiplexing, there is a single eigenmode that gives 10 bits s^−1^ Hz^−1^ at the start of the chain and reducing to 5.75 bits s^−1^ Hz^−1^ after 20 hops. The figure shows that where crosstalk occurs, only the principal eigenmode can carry data along the complete chain and the other eigenmodes become unusable after around 4 hops. Hence, either the crosstalk needs reducing or decode-and-forward relays would be required, which would increase the cost, complexity and latency of the chain. Good agreement is shown between the results obtained from using the measured and modelled crosstalk profiles. However, further improvement would be possible by using a model with more degrees of freedom than the exponential law model used here. Further refinement of the crosstalk model is beyond the scope of this paper.

Figure 6 shows the gain profile of the relay chain, and it is shown that scenarios where crosstalk occurs require additional gain. This is to overcome the reduction in SNR because of the crosstalk and illustrates a trade-off as the additional amplification will also enhance the noise. It also shows the case of no OAM multiplexing, i.e. a single eigenmode requiring additional gain. This is because only a single transmit chain is required compared to four when 4-OAM mode multiplexing is enabled.

The BER profile in figure 7 further illustrates the observations made so far in terms of the impact crosstalk has on performance. It shows that when no crosstalk is present, BER increases from less than 10^−9^ at the start of the relay chain to 10^−2^ after 20 hops. The same is shown for the first mode when crosstalk is present. Eigenmodes 2, 3 and 4 do not exhibit a useful BER past the first hop. This indicates the need for applying well-established error mitigation techniques such as reducing crosstalk, increasing the transmit power, using error correction or interference reduction schemes such as those used in conventional MIMO systems. The use of decode and forward (DF) relays would also have a positive impact on the error performance. DF relays may be used instead of AF relays, or in a hybrid configuration. With the energy requirements and cost of suitable platforms being considerably less than in the past, this becomes a viable option if the added latency is compatible with the system requirements.

The experimental OAM system described in [4] is shown to achieve crosstalk levels of −12.5 dB, whereas the work in [5] reports on a system with below −9.7 dB. In [16], a crosstalk level of better than −13 dB was achieved. None of these studies use partial aperture sampling as an OAM mode filter, which is what has been used for obtaining measured crosstalk here and reported in [9]. Thus, the measured crosstalk levels here may be improved by: using full aperture sampling, careful link alignment and by reducing mutual coupling between antenna elements through careful design. Further investigation is required to determine how much improvement can be expected. Some simple steps can be made, however, as well as experimenting with meta-materials and quasi-optical ways of generating and receiving OAM modes.

It is evident that crosstalk is detrimental to spectral efficiency, and we propose that it can be mitigated by multiplying the output of each receive array with the inverse crosstalk matrix, i.e. ξ^−1^. Good design should also be employed to minimize crosstalk from the outset, i.e. maximize isolation between array feeds for each mode and minimize signal imbalances and multipath. It is also evident that performance degrades as the mode order increases. Consequently, it is proposed that pairs of symmetric modes are used, i.e. ±1, ±2, etc. This is because it would reduce the maximum mode order and hence reduce the maximum null width as well as detrimental effects of mutual coupling because less antenna elements and related RF feeds would be required. Detailed analysis of this is beyond the scope of this paper.

Allocating power across the modes using ‘water-filling’ achieves capacity in theory. However, this scheme requires the transmitter to know the attenuation of each channel so that it can calculate the optimal power levels. Additionally, water-filling can only achieve capacity if it is employed with a fine-grained bit loading scheme, which ensures high-order modulation schemes are used on modes corresponding to high SNR values. Such approaches have been well studied and applied in practice (e.g. ADSL), although the adaptation of these methods for the system considered herein requires further consideration.

## 5. Conclusion

This paper has analysed the spectral efficiency and BER profiles of a relay chain employing OAM multiplexing to support several parallel data streams. The results show that when no crosstalk is present, the spectral efficiency of each of the eigenmodes is identical. However, when crosstalk is present, the spectral efficiencies diverge away from each other. This suggests that crosstalk is detrimental to the system spectral efficiency if not properly accounted for. The crosstalk can be mitigated by employing a non-scalar amplification factor *β* at each of the relays that is equal to the inverse of the crosstalk matrix. Careful mode assignment is also proposed to improve performance. This is necessary to reach useful performance levels.

## Data accessibility

All data required to reproduce this work is contained within the manuscript and associated references.

## References

[1] Simmons D, Coon J, Warsi N 2016 Capacity and power scaling laws for finite antenna MIMO amplify-and-forward relay networks. IEEE Trans. Inf. Theory 30, 1993–2008. (10.1109/TIT.2016.2527682)

[2] Chandra A, Bose C, Kr Bose M 2011 Wireless relays for next generation broadband networks. IEEE Pots 30, 39–43. (10.1109/MPOT.2011.940778)

[3] Drysdale TD, Allen B, Coon JP 2017 Enhanced spectral efficiency of wireless communications alongside transport infrastructure. In IET Conf. A&P for Trans, Birmingham.

[4] Yan Y et al 2014 High-capacity millimetre-wave communications with orbital angular momentum multiplexing. Nat. Commun. 5, 4876 (10.1038/ncomms5876) 25224763 PMC4175588

[5] Zhang W, Zheng S, Hui X, Dong R, Jin X, Chi H, Zhang X 2017 Mode division multiplexing communication using microwave orbital angular momentum: an experimental study. IEEE Trans. Wirel. Commun. 16, 1308–1318. (10.1109/TWC.2016.2645199)

[6] Lee D, Sasaki H, Fukumoto H, Yagi Y, Kaho T, Shiminzu T 2018 An experimental demonstration of 28 GHz band wireless OAM-MIMO (orbital angular momentum multi-input and multi-output) multiplexing. In 87th Vehicular Technology Conf. (VTC Spring), Porto, Portugal, 3–6 June Piscataway, NJ: IEEE.

[7] Tamburini F, Mari E, Sponzelli A, Thidé B, Bianchini A, Romanato F 2012 Encoding many channels on the same frequency through radio vorticity: first experimental tests. New J. Phys. 14, 033001 (10.1088/1367-2630/14/3/033001)

[8] Edfors O, Johansson AJ 2012 Is orbital angular momentum (OAM) based radio communication an unexploited area? IEEE Trans. Antennas Propag. 60, 1126–1131. (10.1109/TAP.2011.2173142)

[9] Drysdale TD, Allen B, Stevens C, Berry SJ, Smith FC, Coon J 2018 How orbital angular momentum modes are boosting the performance of radio links. IET Microw. Antennas Propag. 12, 1625–1632. (10.1049/iet-map.2017.0293)

[10] Zhong X, Zhao Y, Ren G, He S, Wu Z 2018 Influence of finite apertures on orthogonality and completeness of Laguerre–Gaussian beams. IEEE Access 6, 8742–8754. (10.1109/ACCESS.2018.2806887)

[11] Nienhuis G 2017 Analogies between optical and quantum mechanical angular momentum. Phil. Trans. R. Soc. A 375, 20150443 (10.1098/rsta.2015.0443) 28069774

[12] Morabito AF, Di Donato L, Isernia T 2018 Orbital angular momentum antennas: understanding actual possibilities through the aperture antennas theory. IEEE Antennas Propag. Mag. 60, 59–67. (10.1109/MAP.2018.2796445)

[13] Allen B, Drysdale TD, Zhang S, Isakov D, Tennant A, Whittow W, Stevens C, Vardaxoglou J, Coon J 2018 Reduction of orbital angular momentum radio beam divergence using a 3D printed planar graded index lenses. In European Conf. on Antennas and Propagation, London.

[14] Drysdale TD, Allen B 2017 Metal lens for collimation of orbital angular momentum radio modes. In Loughborough Antennas and Propagation Conf., Loughborough.

[15] Veysi M, Guclu C, Capolino F, Rahmat-Samii Y 2018 Revisiting orbital angular momentum beams. IEEE Antennas Propag. Mag. 60, 68–81. (10.1109/MAP.2018.2796439)

[16] Ohtomo I, Yamada K, Nunotani T 1973 Channel multiplexing network for a 20 GHz radio-relay transmission system. IEEE Trans. Microw. Theory Tech. 20, 492–494. (10.1109/TMTT.1973.1128042)

[17] Bai Q, Tennant A, Allen B 2014 Experimental circular phased array for generating OAM radio beams. IET Electron. Lett. 50, 1414–1415. (10.1049/el.2014.2860)

[18] Proakis JG, Salehi M 2001 Communication systems engineering. Upper Saddle River, NJ: Prentice-Hall.

